# The Role of Chinese EMI Teachers’ Clarity and Credibility in Fostering Students’ Academic Engagement and Willingness to Attend Classes

**DOI:** 10.3389/fpsyg.2021.756165

**Published:** 2021-09-17

**Authors:** Jin Zheng

**Affiliations:** School of International Education, Yellow River Conservancy Technical Institute, Kaifeng, China

**Keywords:** teacher clarity, teacher credibility, student academic engagement, student willingness to attend classes, Chinese EMI teachers

## Abstract

Given the fact that students’ absence and disengagement are among the major challenges that teachers experience in educational contexts, investigating factors contributing to students’ academic engagement and willingness to attend classes is of high importance. These challenges are more common in English as a medium of instruction (EMI) classes wherein students are usually not skilled enough to participate in academic tasks and activities. Accordingly, the present study probed into the role of Chinese EMI teachers’ clarity and credibility in fostering students’ academic engagement and willingness to attend classes. In doing so, the E-version of Teacher Clarity Short Inventory, Source Credibility Scale, Utrecht Work Engagement Scale for Students, and Willingness to Attend Classes Questionnaire was virtually distributed among 832 Chinese college students. Performing correlational analyses, strong associations were found between teachers’ clarity and credibility and students’ academic engagement and willingness to attend classes. To assess the power of Chinese EMI teachers’ clarity and credibility in predicting students’ academic engagement and willingness to attend classes, structural equation modeling (SEM) was employed. The results of SEM analysis illuminated that both teacher clarity and credibility are strong predictors of students’ academic engagement and willingness to attend classes. The implications of the findings are also discussed.

## Introduction

Since students’ absence and disengagement may result in academic failure (Janosz et al., 2008; Oluremi, 2014; Wang and Fredricks, 2014), enhancing students’ willingness to attend classes and to become engaged in class activities has always been a concern for teachers in any instructional-learning context. This issue is more important for EMI teachers who instruct academic subjects through English (Macaro et al., 2018; Curle and Derakhshan, 2021). It is mainly due to the fact that most students in EMI classes suffer from inadequate English proficiency, which demotivates them to take part in learning tasks and activities (Soe et al., 2020). Student willingness to attend classes is conceptualized as “their tendency to be psychologically and physically present in the classroom” (Credé et al., 2010, p. 273). Student academic engagement is also defined as “the time and energy that students devote to educationally sound activities” (Kuh, 2009, p. 6). There is a consensus among academics when it comes to the importance of students’ class attendance and academic engagement in their success. Fadelelmoula (2018), for instance, stated that students’ attendance and engagement can highly impact their academic performance. That is, those who regularly attend classes and actively participate in classroom activities will considerably outperform their disengaged and inattentive peers. In a similar vein, Gershenson (2016) also postulated that students’ higher learning outcomes are directly associated with their class attendance and academic involvement. In this regard, identifying factors that increase students’ academic engagement and their willingness to attend classes are extremely important. As put forward by Brewer and Burgess (2005), teachers as the key component of any educational system can dramatically influence the learning environment and student-related variables. Based on this premise, several empirical studies were carried out to explore the impact of teachers’ personal and interpersonal factors on students’ engagement (e.g., Martin et al., 2012; Van Uden et al., 2014; Imlawi et al., 2015; Baralt et al., 2016; Ahmed et al., 2018; Jiang and Zhang, 2021) and willingness to attend classes (e.g., Gershenson, 2016; Liu and Loeb, 2021). However, the effects of teacher clarity and credibility on the aforementioned variables have remained elusive. That is, a few scholars (e.g., Pishghadam et al., 2019; Derakhshan, 2021; Zheng, 2021) endeavored to probe into the role of teacher clarity and credibility in promoting students’ inclination to attend classes and to engage in the learning process. Furthermore, no empirical research has been conducted to investigate the possible associations between these variables in EMI classes. Therefore, the current study intends to fill these research lacunas by examining the role of Chinese EMI teachers’ clarity and credibility in fostering students’ academic engagement and willingness to attend classes.

The construct of teacher clarity, as an instance of teacher interpersonal factor, refers to “the extent to which an instructor presents course content in an understandable and organized manner” (Comadena et al., 2007, p. 242). In a similar vein, Rodger et al. (2007) conceptualized this construct as the capacity of instructors to teach materials in a way that students fully comprehend. As put forward by Zheng (2021), having clarity in classrooms enables teachers to effectively engage their pupils in learning activities. Another teacher interpersonal behavior that may affect student-related variables is teacher credibility, referring to “the degree to which a teacher is believable to students” (Pogue and Ahyun, 2006, p. 332). Teacher credibility as a multidimensional construct encompasses three components of “*Competence*,” “*Trustworthiness*,” and “*Caring*” (Banfield et al., 2006). The components of competence and trustworthiness refer to “the capability of teachers in subject matters” (Derakhshan, 2021, p. 5). Besides, caring, as the last component of teacher credibility, is related to the extent to which teachers value their students’ feelings and pay attention to their interests, desires, and preferences (Pishghadam et al., 2021). Imlawi et al. (2015) proposed that competent and attentive teachers can remarkably enhance students’ tendency to become engaged in the process of learning. In a similar vein, Pishghadam et al. (2021) also suggested those students who perceive their teachers as credible are more inclined to attend their classes.

## Literature Review

### Teacher Clarity

The concept of teacher clarity generally refers to the methods and procedures that teachers employ to ensure that their pupils fully comprehend course materials (Simonds, 1997). In a more detailed conceptualization, Chesebro and McCroskey (2001, p. 62) defined teacher clarity as a construct that depicts the process through which “an instructor is able to effectively stimulate the desired meaning of course content and processes in the minds of students through the use of appropriately-structured verbal and nonverbal messages.” This definition is in line with the idea of instructional communication scholars who believe that teacher clarity as a relational variable has also something to do with the clarity of teaching processes (Finn and Schrodt, 2012; Bolkan, 2016; Linvill and Cranmer, 2017). According to Chesebro and McCroskey’s (2001) conceptualization of teacher clarity, one can reasonably conclude that using appropriate verbal and nonverbal messages is crucial for being a clear teacher. In other words, those instructors who intend to be a clear teacher should employ both verbal and nonverbal cues appropriately. In an attempt to thoroughly characterize clear teachers, Rodger et al. (2007) stated that clear teachers are those who speak fluently, organize course content explicitly, and explain learning subjects effectively.

As put forward by Loes and Pascarella (2015), teacher clarity is closely tied with students’ growth and advancement. That is, those students who experience organized and clear instruction are more successful in mastering the course content (Titsworth et al., 2015). Later, Blaich et al. (2016) stated that the benefits of experiencing organization and clarity in instruction are not limited to mastering the course content and materials. To them, besides students’ achievement and learning outcomes, teacher clarity can also positively affect students’ inclination to engage in learning activities. In light of these premises, some scholars have attempted to probe into the effects of teacher clarity on student-related variables (Finn and Schrodt, 2012; Bolkan, 2016; Violanti et al., 2018). Finn and Schrodt (2012), for instance, explored the association between teacher clarity and student empowerment (i.e., impact, competence, and meaningfulness). In doing so, 261 students were randomly selected from a private college in the southwest. The researchers distributed *Teacher Clarity Scale (TCS)* and *Learner Empowerment Scale (LES)* among participants to gather their viewpoints. Analyzing students’ responses to the aforementioned scales, the researchers found that teacher clarity can remarkably enhance students’ empowerment. In a similar vein, Bolkan (2016) examined the impact of teacher clarity on student learning. To do so, 253 students were invited to complete some pre-developed scales. The analysis of participants’ responses revealed that they perceived teacher clarity as an important predictor of increased learning outcomes.

### Teacher Credibility

The construct of teacher credibility pertains to the extent to which an instructor is believable to his/her pupils. Historically, this concept dates back to “*Rhetoric Theory*” in which Aristotle classified means of persuasion into three main categories, namely, *Pathos*, *Logos*, and *Ethos*. Aristotle defined pathos as “the quality of a persuasive presentation which appeals to the emotions of the audience” (Pishghadam and Karami, 2017, p. 380). He also referred to logos as “the logic used to support a claim” (Nayernia et al., 2020, p. 3). Finally, ethos as the most efficient mode of persuasion is conceptualized as a rhetorical strategy speakers employ to inspire trust in their listeners (Derakhshan, 2021). Aristotle postulated that source credibility is directly related to ethos which encompasses three components: competence, trustworthiness, and caring. Competence refers to the extent to which a teacher is knowledgeable in the eyes of his/her pupils (Zhang, 2009). As the second component, trustworthiness pertains to the degree to which students trust their teachers (Pishghadam et al., 2017). Finally, caring relates to the amount of attention teachers devote to students’ emotions, interests, and desires (Santilli et al., 2011).

To highlight the significance of teacher credibility in educational settings, Wheeless et al. (2011) postulated that a student who perceives his/her teacher as a credible instructor is more inclined to be an active and attentive learner. With regard to such statements, several scholars have tried to empirically investigate the positive consequences of teacher credibility in classroom contexts. Among them, a noticeable number of studies have been dedicated to the impact of teacher credibility on student-related factors (e.g., Gray et al., 2011; Zhang, 2011; Klebig et al., 2016; Ledbetter and Finn, 2018; Amiryousefi and Mirkhani, 2019; Karimi and Ziaabadi, 2019; Pishghadam et al., 2019, 2021; Lee, 2020). Lee (2020), for instance, explored the impact of teacher credibility on students’ willingness to communicate (WTC) in the classroom. To do this, 252 Korean students were randomly selected from a public university. Some valid and reliable questionnaires were distributed among participants to gather their personal beliefs about the facilitative/debilitative role of EFL teachers’ credibility in students’ tendency to communicate in English. Correlation coefficients and multiple regressions were performed to analyze the obtained data. The results illuminated that teacher credibility was perceived as a strong predictor of students’ WTC. That is, credible instructors are more capable of inspiring students to communicate. By the same token, Pishghadam et al. (2021) studied the relationship between EFL teachers’ credibility and their students’ willingness to attend classes. To do so, two valid instruments of “*Teacher Credibility Scale* (TCS)” and “*Willingness to Attend Classes Scale* (WTACS)” were given to 426 EFL students (i.e., 276 Iranian and 150 Iraqi) to gather their viewpoints. The results demonstrated that both Iranian and Iraqi students considered teacher credibility as a significant antecedent of students’ willingness to attend classes.

### Student Academic Engagement

The construct of student academic engagement as a complex and multidimensional factor has been conceptualized in different ways (Balwant, 2018). In their study, Hu and Kuh (2002, p. 556) defined student academic engagement as “the quality of the effort students themselves devote to educationally purposeful activities that contribute directly to the desired outcomes.” Kuh (2003) further referred to this construct as the amount of time and energy students allocate to educational tasks and activities both in and out of the classrooms. The concept of student academic engagement was recently defined by Hiver et al. (2021, p. 2) as “the amount (quantity) and type (quality) of learners’ active participation and involvement in a learning activity.” In an effort to characterize different facets of student academic engagement, Schaufeli et al. (2002) divided this construct into three main dimensions of “*Vigor*, *Absorption*, and *Dedication*.” Vigor, as the first dimension, refers to the amount of effort and perseverance students exhibit in difficult conditions. Absorption also relates to students’ immersion in learning activities. As the last dimension, dedication pertains to students’ inspiration and passion for learning course content (Alrashidi et al., 2016).

Given the pivotal role that students’ academic engagement plays in their success (Carver et al., 2021; Khajavy, 2021), several studies have been conducted to uncover internal and external factors that encourage students to engage in class activities (e.g., Alvandi et al., 2015; Estepp and Roberts, 2015; Rashid and Asghar, 2016; Wong et al., 2017; Zhen et al., 2017; Ahmed et al., 2018; Maricuțoiu and Sulea, 2019; Pedler et al., 2020; Derakhshan, 2021; Jiang and Zhang, 2021). For instance, Estepp and Roberts (2015) probed into the role of teacher-student rapport and teacher immediacy in improving students’ engagement. To do so, 306 university students were asked to fill out three pre-developed questionnaires. Employing canonical correlation analyses, the gathered data were analyzed. The results of analyses depicted that both immediacy and teacher-student rapport were significant predictors of student engagement. Similarly, Alvandi et al. (2015) explored the impact teacher critical thinking skills on students’ engagement. To do this, 600 EFL students voluntarily completed two close-ended questionnaires. The inspection of the correlations revealed a strong connection between teacher critical thinking skills and students’ academic engagement. By the same token, Derakhshan (2021) endeavored to examine the power of teachers’ nonverbal immediacy and credibility in predicting students’ engagement. To this aim, the electronic version of three validated questionnaires was sent to 503 Iranian students. Performing structural equation modeling (SEM), the predictability power of teacher credibility and nonverbal immediacy was assessed. The proposed model illuminated that students’ engagement can favorably be predicted by teachers’ credibility and nonverbal immediacy. It means that credible and immediate teachers are more successful in fostering students’ engagement.

### Student Willingness to Attend Classes

Class attendance is simply defined as students’ inclination to be psychologically and physically present in their classes (Credé et al., 2010). This definition implies that pupils can be physically present in the classroom while their thoughts are elsewhere. Hence, attentive students are those whose presence is not merely physical (Oluremi, 2014). As put forward by Bijsmans and Schakel (2018), students’ regular presence can favorably influence their academic performance. Gershenson (2016) suggested that students’ (dis)inclination to attend classes is extremely dependent on teacher behaviors. To him, those teachers who employ appropriate interpersonal behaviors in interacting with their pupils are more successful in encouraging them to attend their classes. In line with this premise, some researchers have attempted to explore the impact of teacher interpersonal variables on students’ decision to attend classes (e.g., Alhassan and Odame, 2014; Adebisi and Lawal, 2016; Rajabnejad et al., 2017; Pishghadam et al., 2019, 2021; Liu and Loeb, 2021). For instance, Rajabnejad et al. (2017) have sought to investigate the role of teacher stroke in enhancing students’ willingness to attend classes. To this end, they distributed Stroke Scale (SS) and WTAC Scale among 260 Iranian EFL students. Evaluating students’ responses to the questionnaires revealed that those students who received strokes were more inclined to attend their classes. Hence, teacher stroke was identified as an important predictor of students’ willingness to attend classes. In a similar study, Pishghadam et al. (2019) examined the impact of teacher stroke and credibility on students’ tendency to attend classes. In doing so, 276 undergraduate students voluntarily took part in this study. Employing three valid and reliable scales, students’ viewpoints toward the associations between the aforementioned variables were elicited. The results represented that students who perceived their instructors as knowledgeable and trustworthy were more inclined to be present in their courses. Teacher stroke was also identified as a motivational factor that enhances students’ willingness to attend classes.

So far, numerous studies have focused on the positive consequences of teacher clarity and credibility in classroom contexts. Nevertheless, the role of these interpersonal variables in EMI classes has been neglected. Furthermore, not much attention has been paid to the impact of teacher clarity and credibility on students’ academic engagement and willingness to attend classes. To fill the existing gaps in the literature, the current empirical study aims at investigating the role of Chinese EMI teachers’ clarity and credibility in fostering students’ academic engagement and willingness to attend classes.

## Theoretical Underpinning

### Rhetorical-Relational Goal Theory

The main premise of rhetorical-relational goal theory is that teachers have a range of rhetorical and relational objectives that they wish to attain in educational contexts (Mottet et al., 2006). Employing different rhetorical (e.g., clarity and credibility) and relational (e.g., nonverbal immediacy and confirmation) communication behaviors, teachers can provide an affective learning atmosphere wherein students can easily acquire the course content (Myers, 2008; Myers et al., 2018; Xie and Derakhshan, 2021). In this regard, Beebe and Mottet (2009) also stated that the rhetorical and relational behaviors that teachers utilize in their classes enable them to influence their pupils’ academic behaviors, including motivation, commitment, and academic engagement.

## Research Questions

1. Are there any statistically significant associations between Chinese EFL teachers’ clarity and credibility subscales and students’ academic engagement and willingness to attend classes?
2. Do Chinese EMI teachers’ clarity and credibility significantly predict students’ academic engagement and willingness to attend classes?

## The Current Study Method

### Participants

According to the previous studies and some gaps to be filled in the relevant literature in the domain of interpersonal variables in EMI, a total number of 832 EMI students were randomly selected from seven vocational education institutes of four provinces in China (i.e., Jiangsu, Zhejiang, Hubei, and Henan). To maximize the variation of the sample that enhances the generalizability of the outcomes (Ary et al., 2018), the participants were selected from both genders (*N*=356 females and 476 males, respectively), different age levels, and eight different majors covering *Water Conservancy* (168), *Civil Engineering* (179), *Mechanical Process* (86), *Tourism Management* (183), *International Trade* (82), and *Business Management* (134). The respondents who were reassured that their information would be kept secret and be utilized only for research purposes signed their consent agreement before they participated in this survey.

### Instruments

#### Teacher Clarity Short Inventory

The 10-item version of Teacher Clarity Short Inventory (TCSI; Chesebro and McCroskey, 1998) was utilized to assess Chinese EMI teachers’ clarity. The participants’ responses to the items can vary on a 5-point Likert-type scale, from 1 (strongly disagree) to 5 (strongly agree). The reliability index of TSCI was reported as 0.92 (Chesebro and McCroskey, 2001). In the current study, the alpha reliability of TCSI was 0.71.

#### Source Credibility Scale

Chinese EMI teachers’ credibility was measured *via* McCroskey and Teven’s (2013) Source Credibility Scale (SCS). This scale includes 18 items to which respondents answer on a linear scale. The SCS encompasses three subscales, including “*Competence*,” “*Goodwill*,” and “*Trustworthiness*.” These subscales’ reliability has been reported as 0.89, 0.93, and 0.83, respectively (Nayernia et al., 2020). The reliability of SCS was estimated to be 0.86 in this study.

#### Utrecht Work Engagement Scale for Students

To measure Chinese students’ academic engagement, Utrecht Work Engagement Scale for Students (UWES-S) designed by Schaufeli et al. (2002) was employed. This 14-item questionnaire comprises three components of “*Vigor*” (items 1–5), “*Dedication*” (items 6–10), and “*Absorption*” (items 11–14). The UWES-S uses a 7-point Likert scale, varying in responses from 0 (strongly disagree) to 6 (strongly agree). The questionnaire’s reliability has been demonstrated as 0.89 (Derakhshan, 2021). The alpha reliability of the UWES-S in this study was estimated as 0.93, which indicates acceptable reliability.

#### Willingness to Attend Classes Questionnaire

Chinese students’ willingness to attend classes was assessed *via* Willingness to Attend Classes Questionnaire (WTAC) developed and validated by Rajabnejad et al. (2017). The WTAC contains 25 5-point Likert scale items, varying from 0 (totally disagree) to 4 (totally agree). It encompasses five subscales, namely, “*Teacher Knowledge*,” “*Teacher Methodology*,” “*Teacher Care*,” “*Teacher Characteristics*,” and “*Teacher Environment*.” While the reliability index of this measure has been previously computed as 0.88 (Pishghadam et al., 2019), the estimated reliability for this study was 0.94.

### Data Collection Procedure

Prior to initiating the data collection process, the electronic version of consent forms was sent to participants to gather their consent. In order to collect students’ viewpoints, the E-versions of the questionnaires were generated through Wenjuanxing software. Then, the link to the questionnaires was shared with participants *via* WeChat messenger. The respondents were given adequate instructions on how to fill out the questionnaires.

### Data Analysis

First, the Kolmogorov-Smirnov (K-S) test was run to assess the normality of the data. Second, to measure the reliability of the questionnaires, Cronbach’s alpha was employed. Then, to examine the associations between teachers’ clarity and credibility subscales and students’ academic engagement and willingness to attend classes, the Pearson correlation procedure was performed *via* SPSS software (version 27). Further, to identify the impact of Chinese EMI teachers’ clarity and credibility on students’ academic engagement and willingness to attend classes, SEM was utilized.

## Results

As mentioned above, the K-S test was first performed to determine the normality of data. Table 1 depicts the results of K-S test.

**Table 1:** The results of K-S test.

Scale	Kolmogorov-Smirnov		
	Statistic	df	Sig.
TCSI	0.05	832	0.08
SCS	0.06	832	0.07
UWES-S	0.06	832	0.06
WTAC	0.09	832	0.15

As Table 1 demonstrates, the Sig values of the K-S test for teacher clarity, teacher credibility, student academic engagement, and student willingness to attend classes are 0.08, 0.07, 0.06, and 0.15, respectively. As all the Sig values are larger than 0.05, it can be inferred that the distribution of data is normal; hence, parametric tests can be employed. The descriptive statistics of TCSI, SCS, UWES-S, and WTAC are portrayed in Table 2.

**Table 2:** Descriptive statistics of the scales.

Scale	*N*	Minimum	Maximum	Mean	SD
TCSI	832	14.00	50.00	39.05	6.26
SCS	832	24.00	126.00	106.95	11.68
UWES-S	832	14.00	98.00	77.18	8.40
WTAC	832	25.00	125.00	95.13	7.31

Table 3 indicates the results of the Cronbach alpha analyses. According to Table 3, all scales and subscales achieved acceptable indices of Cronbach alpha.

**Table 3:** Results of Cronbach alpha indexes.

Scale	Subscales	Cronbach alpha
TCSI		0.71
SCS	Competence	0.82
	Goodwill	0.79
	Trustworthiness	0.85
	Overall scale	0.86
UWES-S	Vigor	0.91
	Dedication	0.94
	Absorption	0.87
	Overall scale	0.93
WTAC	TK	0.93
	TM	0.84
	TC	0.88
	TCH	0.89
	TE	0.95
	Overall scale	0.94

Further, Pearson correlation was performed to examine the associations between teachers’ clarity and credibility subscales and students’ academic engagement and willingness to attend classes (Table 4).

**Table 4:** Results of Pearson correlation between overall teachers’ clarity and credibility and students’ academic engagement and willingness to attend classes.

Variables		Clarity	Credibility	Academic engagement	WTAC
Clarity	Pearson correlation	1			
	Sig. (two tailed)				
	*N*	832			
Credibility	Pearson correlation	0.58^**^	1		
	Sig. (two tailed)	0.000			
	*N*	832	832		
Academic engagement	Pearson correlation	0.39^**^	0.38^**^	1	
	Sig. (two tailed)	0.000	0.000		
	*N*	832	832	832	
Willingness to attend classes		0.37^**^	0.35^**^	0.72^**^	1
		0.000	0.000	0.000	
		832	832	832	832

** *Correlation is significant at the 0.01 level (two tailed)*.

As shown in Table 4, strong associations were found between overall teacher clarity and credibility (*r*=0.58, *n*=832, *p*=0.000, *α*=0.01), student academic engagement (*r*=0.39, *n*=832, *p*=0.000, *α*=0.01), and student willingness to attend classes (*r*=0.37, *n*=832, *p*=0.000, *α*=0.01). Furthermore, statistically significant correlations were also found between overall teacher credibility and student academic engagement (*r*=0.38, *n*=832, *p*=0.000, *α*=0.01), and student willingness to attend classes (*r*=0.35, *n*=832, *p*=0.000, *α*=0.01). Finally, Pearson correlation revealed a positive correlation between student academic engagement and their willingness to attend classes (*r*=0.72, *n*=832, *p*=0.000, *α*=0.01).

**Figure 1:**
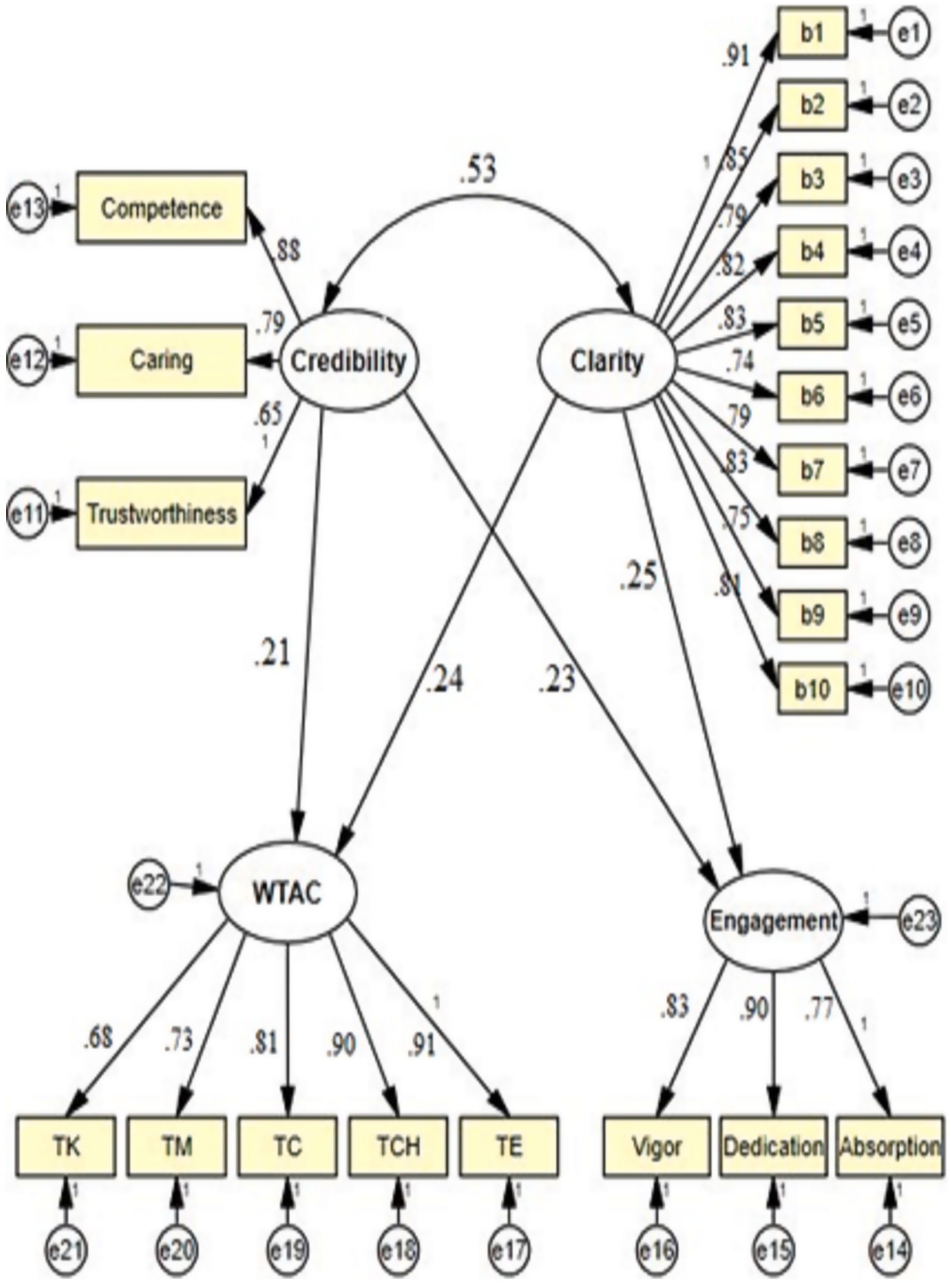
The structural equation model of the interrelationships among teacher clarity and credibility and student academic engagement and willingness to attend classes.

Structural equation modeling was further employed to examine the power of Chinese EMI teachers’ clarity and credibility in predicting students’ academic engagement and willingness to attend classes. Figure 1 depicts the model of the interrelationships among the aforementioned variables.

As Figure 1 delineates, both teacher clarity (*β*=0.25, *p*<0.05) and credibility (*β*=0.23, *p*<0.05) were found as the strong antecedents of students’ academic engagement. Moreover, students’ willingness to attend classes was also favorably and significantly predicted by both clarity (*β*=0.25, *p*<0.05) and credibility (*β*=0.23, *p*<0.05).

Multiple fit indices, including X2/df, GFI, CFI, NFI, and RMSEA, were computed to determine whether the given data fit the proposed model (Table 5). To have a fit structural equation model, RMSEA should be less than 0.08, df should be less than 3, and GFI, CFI, and NFI should be more than 0.90.

**Table 5:** Goodness of fit indices.

	X2/df	GFI	CFI	NFI	RMSEA
Acceptable fit	<3	>0.90	>0.90	>0.90	<0.08
Model	2.77	0.93	0.95	0.92	0.06

As Table 5 demonstrates, all fit indices are within the acceptable range, indicating that the proposed model provided an acceptable fit with the obtained data.

## Discussion

The current study was primarily aimed at assessing the interrelationships among Chinese EMI teachers’ clarity and credibility and students’ academic engagement and willingness to attend classes. The results of correlation analyses illuminated a strong association between both teacher clarity and credibility and students’ willingness to attend classes. A statistically significant association was also found between teacher clarity and credibility and students’ academic engagement. The association between teacher clarity and students’ willingness to attend classes can be reasonably justified by the fact that those students who experience organized and clear instruction are naturally more inclined to attend their classes (Blaich et al., 2016). Moreover, the relationship between teacher credibility and students’ willingness to attend classes can also be clarified by the rhetorical-relational goal theory proposed by Mottet et al. (2006), which links teachers’ rhetorical behaviors, such as credibility with students’ academic behaviors.

Furthermore, the connection between teacher clarity and students’ academic engagement can be easily explicated by the fact that those students who receive organized and clear instructions are more inclined to become involved in the learning process (Brewer and Burgess, 2005). Finally, the correlation between teacher credibility and students’ academic engagement may be explained by the fact that knowledgeable and trustworthy instructors are more successful in inspiring students to engage in classroom activities (Wheeless et al., 2011).

The present empirical study was also intended to examine the power of Chinese EMI teachers’ clarity and credibility in predicting students’ academic engagement and willingness to attend classes. The results of SEM analysis illuminated that students’ academic engagement is favorably predicted by both teacher clarity and credibility. This finding confirms the ideas of Zheng (2021), who postulated that positive interpersonal behaviors, such as clarity and credibility that teachers employ in instructional-learning contexts, inspire students to take part in educational activities. The predictability of students’ academic engagement through teachers’ credibility is in agreement with Derakhshan’s (2021) findings which demonstrated that Persian language teachers’ credibility can positively influence their students’ academic engagement. This result is also consistent with those of Imlawi et al. (2015), who found that competent and attentive teachers can foster students’ academic engagement.

Like academic engagement, students’ willingness to attend classes is also positively and remarkably predicted by both teacher clarity and credibility. The power of teacher clarity in predicting students’ willingness to attend classes can be illustrated by the ideas of Gershenson (2016) who stated that clear teachers who are able to effectively organize and instruct course content can remarkably impact students’ inclination to be psychologically and physically present in their classes. In addition, the positive effects of teacher credibility on students’ willingness to attend classes can be justified by students’ natural tendency to have competent and trustworthy instructors. This finding is in agreement with those of Pishghadam et al. (2021), who found teacher clarity as a strong antecedent of students’ willingness to attend classes.

## Conclusion

The results of the current study culminated in an important theme, namely, both Chinese EMI teachers’ clarity and credibility can positively and dramatically predict students’ academic engagement and willingness to attend classes. To put it simply, those students who receive instructions from a clear and credible teacher are more likely to attend classes and become involved in the learning process. These findings have some important implications for teachers in any educational context, notably EMI classes. Students’ absence and disengagement are among the important challenges that teachers typically experience in classroom contexts. Based on the findings of this study, those teachers who intend to enhance their students’ tendency to attend classes and to take part in classroom activities should instruct course content clearly. Moreover, being considered credible enable instructors to motivate their pupils to become attentive and active learners. Hence, teachers are strongly advised to enhance their credibility in educational settings. These findings can also be illuminating for teacher trainers. Given the pivotal role that teacher clarity and credibility play in promoting students’ academic engagement and willingness to attend classes, teacher trainers should instruct teachers on how to enhance their credibility and instructional clarity in classroom contexts.

Finally, a number of important limitations need to be enumerated. The first limitation lies in the fact that the current study was conducted in EMI classes; hence, the findings might not be transferable to other educational contexts. To discover any discrepancy in the findings, further research is recommended to replicate this inquiry in other instructional-learning contexts. Second, only closed-ended questionnaires were employed to collect the participants’ viewpoints. To obtain a deeper insight into the topic, further empirical investigations are needed to include some open-ended questionnaires and structured/semi-structured interviews. Third, the probable effects of situational factors, such as gender, age, and major, were not considered. Future works are advised to control or measure the impact of these factors. Furthermore, there seems to be a desideratum to scrutinize the interplay between teacher-student interpersonal factors and dynamic variables subsumed under the positive psychology in SLA (Wang et al., 2021; Wang and Derakhshan, 2021).

## Data Availability Statement

The original contributions presented in the study are included in the article/supplementary material, and further inquiries can be directed to the corresponding author.

## Ethics Statement

The studies involving human participants were reviewed and approved by the Education Affairs Office and Academic Ethics Committee by Yellow River Conservancy Technical Institute. The patients/participants provided their written informed consent to participate in this study.

## Author Contributions

JZ, as the corresponding author, conceived the initial idea, designed the study, collected the data, analyzed the data, and drafted the manuscript.

## Funding

This study was supported by the Foreign Language Education Teaching Project of Vocational Colleges of the Ministry of Education in 2020 – “Vocational English Teaching Model Based on Knowledge Graph and Progression” (no. WYJZW-2020-1357) and the project of Yellow River Conservancy Technical Institute – “Research on Self-Adaptive Teaching Model of Public English from the Perspective of Multiple-level students” (no. 2020XJJGLXYB008).

## Conflict of Interest

The author declares that the research was conducted in the absence of any commercial or financial relationships that could be construed as a potential conflict of interest.

## Publisher’s Note

All claims expressed in this article are solely those of the authors and do not necessarily represent those of their affiliated organizations, or those of the publisher, the editors and the reviewers. Any product that may be evaluated in this article, or claim that may be made by its manufacturer, is not guaranteed or endorsed by the publisher.
